# Reprogramming of Melanoma Tumor-Infiltrating Lymphocytes to Induced Pluripotent Stem Cells

**DOI:** 10.1155/2016/8394960

**Published:** 2015-12-28

**Authors:** Hidehito Saito, Keisuke Okita, Noemi Fusaki, Michael S. Sabel, Alfred E. Chang, Fumito Ito

**Affiliations:** ^1^Department of Surgery, University of Michigan, 1500 E Medical Center Drive, 3410 CC, Ann Arbor, MI 48109-5932, USA; ^2^Center for iPS Cell Research and Application, Kyoto, Japan; ^3^Department of Ophthalmology, Keio University School of Medicine, Tokyo, Japan; ^4^DNAVEC Corporation, Tsukuba, Ibaraki, Japan; ^5^Department of Cell and Developmental Biology, University of Michigan, Ann Arbor, MI 48109-2200, USA

## Abstract

Induced pluripotent stem cells (iPSCs) derived from somatic cells of patients hold great promise for autologous cell therapies. One of the possible applications of iPSCs is to use them as a cell source for producing autologous lymphocytes for cell-based therapy against cancer. Tumor-infiltrating lymphocytes (TILs) that express programmed cell death protein-1 (PD-1) are tumor-reactive T cells, and adoptive cell therapy with autologous TILs has been found to achieve durable complete response in selected patients with metastatic melanoma. Here, we describe the derivation of human iPSCs from melanoma TILs expressing high level of PD-1 by Sendai virus-mediated transduction of the four transcription factors, OCT3/4, SOX2, KLF4, and c-MYC. TIL-derived iPSCs display embryonic stem cell-like morphology, have normal karyotype, express stem cell-specific surface antigens and pluripotency-associated transcription factors, and have the capacity to differentiate *in vitro* and *in vivo*. A wide variety of T cell receptor gene rearrangement patterns in TIL-derived iPSCs confirmed the heterogeneity of T cells infiltrating melanomas. The ability to reprogram TILs containing patient-specific tumor-reactive repertoire might allow the generation of patient- and tumor-specific polyclonal T cells for cancer immunotherapy.

## 1. Introduction

A groundbreaking discovery showing that differentiated somatic cells can be reprogrammed by transiently overexpressing a defined set of transcription factors offers the opportunity to obtain patient-specific somatic cells for potential therapeutic applications [1–4]. These reprogrammed cells, referred to as induced pluripotent stem cells (iPSCs), can be generated from mature peripheral blood T cells [5–8]. T cell derived iPSCs can redifferentiate into functional CD8^+^ T cells harboring long telomeres and increased proliferative capacity [9, 10]. These “rejuvenated human T cells” exhibit T cell receptor (TCR) gene rearrangement patterns identical to the parental T cells, from which the iPSC clones were established, and antigen-specific killing effector functions* in vitro* [9–11]. Moreover, the iPSCs engineered to express TCR of known antigen specificity can differentiate to antigen-specific T cells, promote cancer immunosurveillance, and mediate antitumor immunity* in vivo* [12, 13]. These findings suggest possible applications of iPSCs for use as a cell source for producing lymphocytes for cell-based therapy against cancer.

Adoptive cell therapy with autologous tumor-infiltrating lymphocytes (TILs) has emerged as one of the most effective treatments for patients with metastatic melanoma. A major limitation of this approach is poor survival of T cells* in vivo* following infusion. The majority of TILs are terminally differentiated effector T cells that express high levels of immunoinhibitory receptors such as programmed cell death protein-1 (PD-1), indicative of the “exhausted phenotype” and functional impairment [14–16]. Current clinical protocols for adoptive T cell therapy stipulate that differentiated T cells require further stimulation in order to obtain large numbers of T cells. This results in generation of terminally differentiated CD8^+^ T cells that exhibit decreased antitumor efficacy* in vivo* due to their diminished capacity to maintain effector function after infusion compared with less-differentiated CD8^+^ T cells [17–23]. This limitation of adoptive T cell therapy can be overcome by using iPSCs that self-renew, maintain pluripotency [1–4], and provide an unlimited source of autologous polyclonal T cells for treating heterogeneous tumors. However, the differentiation status of the donor cell is known to influence the efficiency of embryonic cell (ESC) derivation as well as iPSC generation [24, 25]. Hence, the feasibility of reprogramming terminally differentiated and exhausted TILs remains unknown.

Here, we report successful generation of human iPSCs from terminally differentiated melanoma TILs that express high levels of PD-1 by Sendai virus- (SeV-) mediated transduction of the four transcription factors OCT3/4, SOX2, KLF4, and c-MYC. All of the iPSCs generated from TIL culture using SeV reprogramming system have TCR rearranged genes indicating that they are derived from mature T cells. Detection of a wide variety of TCR gene rearrangement patterns in TIL-iPSCs is indicative of heterogeneous T cell populations in melanoma TILs.

## 2. Materials and Methods

### 2.1. Ethics Statement

The study was approved by the Institutional Review Board (IRB) of the University of Michigan (protocol number HUM00054459) and the Human Pluripotent Stem Cell Research Oversight (HPSCRO) Committee (protocol number 1055) and has been performed in accordance with the ethical standards of the responsible committee on human experimentation and with the Helsinki Declaration. An IRB-approved written informed consent was obtained from all patients for being included in the study.

All animal care and procedures were in accordance with institutional policies for animal health and well-being and approved by the University Committee on Use and Care of Animals (UCUCA) at the University of Michigan under protocol number PRO00005921. Mice were euthanized using CO_2_ and cervical dislocation according to the University of Michigan UCUCA guidelines.

### 2.2. Patient Cell Samples

Patients were eligible for this study if they were 18 years of age or older and were undergoing resection of metastasis and/or regional lymph node dissection for clinically evident regional or distant metastases of melanoma. Patients with an immunosuppressive disorder or an autoimmune disorder or patients receiving treatment with immunosuppressive drugs for melanoma within 3 months prior to entry into the study were excluded.

After written informed consent, an up to 30 mL sample of venous blood was collected in EDTA tubes (BD Biosciences) from the patients. Peripheral blood mononuclear cells (PBMCs) were isolated using Ficoll-Paque (GE Healthcare Life Sciences) density-gradient centrifugation. Freshly resected tumors were sent from the surgery suite to the tissue procurement core in sterile containers. The tumor material not required for histopathologic diagnosis was placed in collecting medium, RPMI 1640 supplemented with 200 *μ*g/mL gentamicin, 5 *μ*g/mL ciprofloxacin, 20 *μ*g/mL metronidazole, 100 U/mL penicillin, 50 *μ*g/mL streptomycin, 25 *μ*g/mL vancomycin, and 2.5 *μ*g/mL fungizone (all from Life Technologies). Under sterile conditions, tumors were dissected away from adjacent normal tissue and stroma.

### 2.3. Mice

Severe combined immunodeficient female mice 6–8 weeks old (CbySmn.CB17-Prkdc^scid^/J SCID) were obtained from Jackson Laboratory. Mice were maintained in pathogen-free barrier conditions.

### 2.4. Generating TIL Cultures

Single-cell suspensions were obtained by mechanical dispersion consisting of two 30 min incubations of 2.5 g minced melanoma tumor at 37°C and 5% CO_2_ in 5 mL RPMI 1640 (Life technologies) and tumor dissociation kit (Miltenyi Biotec) in C Tubes (Miltenyi Biotec) interspersed with three mechanical dispersions on a GentleMACS dissociator (Miltenyi Biotec). The tumor cell suspensions were then filtered through a cell strainer (75 *μ*m, BD Biosciences). TILs and tumor cells were isolated with a two-step density-gradient centrifugation [26]. In brief, tumor suspension in RPMI was layered onto a two-step gradient with a lower step of 100% Ficoll and a middle step of 75% Ficoll and 25% complete medium. After 45 minutes' centrifugation at 400 g, the interfaces were collected. The lower, TIL-enriched fraction was cultured in 24-well plates in 2 mL T cell media containing IMDM supplemented with 6,000 IU/mL recombinant human (rh) IL-2 (Aldesleukin, Prometheus Laboratories Inc.), 10% human serum, 2 mmol/L l-glutamine, 100 U/mL penicillin, 100 *μ*g/mL streptomycin, 500 *μ*g/mL gentamicin, and 55 *μ*M 2-mercaptoethanol (all from Life Technologies) or collected for flow cytometric analysis. Half of the medium was changed on day 5 after culture initiation and every 2-3 days thereafter. TILs were split 1 : 2, doubling the number of wells when reaching confluence. TILs were cryopreserved after expansion in 10% DMSO in human serum.

### 2.5. Generation of iPSCs from Melanoma TILs

After clearance of adherent cells and a week in log phase expansion (days 21 to 28 after initiation of cultures), TILs were activated with anti-CD3/CD28 (BD Biosciences) and rhIL-2 (6,000 IU/mL) for 5 days. Then, TILs were reactivated with anti-CD3/CD28 in reprogramming media, X-VIVO 15 medium (Lonza) containing 5% FBS, 20 mM HEPES, 2 mM L-glutamine, and 10 U/mL of penicillin, 100 *μ*g/mL of streptomycin (all from Life Technologies), 10 mM N-acetylcysteine (Cumberland pharmaceuticals), and 60 IU/mL rhIL-2 for 24 hours in 24-well plate at 1 × 10^5^ cells/well. Then, TILs were infected with SeV vectors that individually carried each of OCT3/4, SOX2, KLF4, and c-MYC at various multiplicity of infection (MOI). In some experiments, TILs were infected with SeV encoding green fluorescent protein (SeV-GFP) to determine transduction efficiently. After 24 hours of infection, the cells were collected and transferred to a 10 cm dish that contained mitomycin C-inactivated SNL feeder cells (Cell Biolabs, Inc.) in hESC media which consisted of Primate ES Cell Medium (Reprocell) and 4 ng/mL basic fibroblast growth factor (bFGF, Life technologies). The hESC medium was changed every other day until the colonies were picked. Then, the hESC culture medium was changed every day and the cells were passaged using 1 mg/mL collagenase IV every 5-6 days. H1-ESCs (WiCell) were used as controls for characterizing pluripotency of generated iPSCs.

### 2.6. Flow Cytometry

The following mAbs specific for human antigens and appropriate isotype controls were used: from BD Biosciences, allophycocyanin-Cy7-conjugated anti-CD8 (RPA-T8), PE anti-CD4 (RPA-T4), PerCP anti-TIM-3 (F38-2E2), PE/Cy7 anti-LAG-3 (3DS223H), Alexa Fluor anti-CD45RO (UCHL1), and allophycocyanin anti-CCR7 (150503); and from eBioscience, FITC anti-PD-1 (J105). Cells were resuspended in staining buffer (PBS containing 2% FBS) and were stained with mAb against surface Ags for 30 min at 4°C in the dark. Acquisition of at least 10,000 events was done on LSR II flow cytometer (BD Biosciences). Cell aggregates and dead cells were excluded by forward and side scatter and with DAPI staining for unfixed cells. Flow cytometry analysis was carried out with FlowJo version 10.0.7 software (Tree Star, Ashland, OR), gating based on isotype control Ab staining, and the number in each gate represents the percentage of cells.

### 2.7. Alkaline Phosphatase (ALP) and Immunofluorescence Staining of iPSCs

ALP staining was performed with the ALP substrate (Sigma) after fixation with 4% paraformaldehyde. Immunofluorescence staining was performed using the following primary antibodies: anti-OCT 3/4 (sc-5279, Santa cruz), anti-SSEA3 (MAB4303, Millipore), anti-SSEA4 (MAB4304, Millipore), anti-TRA-1-60 (MAB4360, Millipore), and anti-TRA-1-81 (MAB4381, Millipore). The fluorescence signals were detected using a conventional fluorescence laser microscope equipped with a color charge-coupled device (CCD) camera. The secondary antibodies used were anti-mouse IgG and IgM or anti-rat IgM conjugated with Alexa Fluor 488 (Jackson Immunoresearch).

### 2.8. Gene Expression Analysis by Reverse Transcription-Polymerase Chain Reaction (RT-PCR)

Total RNA samples were isolated with TRIZOL reagent (Life Technologies) from iPSCs and H1-ESCs and purified, according to the manufacturer's instructions. One microgram total RNA was used for cDNA synthesis by RT-PCR with SuperScript III Reverse Transcriptase (Life Technologies) and random primer, according to the manufacturer's instructions. cDNA was amplified by PCR with Platinum* Taq* DNA Polymerase (Life Technologies) and various sets of primers shown in Table S1 in Supplementary Material available online at http://dx.doi.org/10.1155/2016/8394960.

### 2.9. Global Gene Expression Analysis

To compare transcriptomes of ESCs and iPSCs, microarray analysis was performed. RNA sample processing, probing to the human gene 2.1 ST array platform (Affymetrix), and data analysis were performed by the University of Michigan DNA Sequencing Core Microarray Facility. Normalized expression by robust multiple-array averages was plotted using the heatmap.2 function from the g-plots package in R (http://www.r-project.org) using default parameters. The euclidean distance dissimilarity matrix and complete linkage method were used to generate the dendrogram.

### 2.10. *In Vitro *Differentiation of iPSCs

For the embryoid body (EB) formation, iPSCs were harvested by collagenase IV and transferred to ultralow attachment plates in hESC medium without bFGF. After 8 days, aggregated cells were plated onto gelatin-coated dishes for another 8 days. The cells were fixed with PBS containing 4% paraformaldehyde for 15 minutes at room temperature. Immunostaining was performed as described above. The primary antibodies used were an anti-Sox17 antibody (1 : 200, R&D Systems), anti-SMA antibody (1 : 500, 1A4, DAKO), and anti-*β*III tubulin (1 : 1000, TUJ1, Covance). The secondary antibodies used were Alexa Fluor 488-conjugated donkey anti-Goat IgG (1 : 500, Life Technologies) for Sox17 and Alexa Fluor 488-conjugated donkey anti-mouse IgG (1 : 500, Life Technologies) for SMA and *β*III tubulin. DAPI (Molecular Probes) was used for nuclear staining.

### 2.11. Teratoma Formation and Histological Analysis

Undifferentiated TIL-derived iPS cells (1 × 10^6^) suspended in 100 *μ*L of DMEM containing 10% FCS were injected into the subcutaneous tissue of 6- to 8-week-old female NOD/SCID mice. Six weeks after injection, tumors were surgically dissected from the mice. Samples were fixed in PBS containing 10% paraformaldehyde and embedded in paraffin. Paraffin-embedded samples were stained with hematoxylin and eosin for histological analysis.

### 2.12. T Cell Receptor-*β* Chain Rearrangement Analysis

Genomic DNA was isolated per manufacturer's protocol (using the Qiagen DNeasy Blood and Tissue kit) from the TIL-iPS cell lines. TCR clonotype mapping was performed using a kit for analyzing T cell receptor-*β* chain rearrangements (InVivoScribe Technologies). PCR was performed using a multiplex primer kit (InVivoScribe Technologies) specific for a majority of clonal TCR-*β* chain rearrangements [27]. Capillary electrophoresis and PCR product fragment analysis was performed at the University of Michigan Sequencing Core Facility (Ann Arbor, MI) using an ABI 3730 DNA analyzer. Data was analyzed using Peak Scanner software (Applied Biosystems).

### 2.13. Karyotyping

Standard G-banded karyotyping was carried out and interpreted by Cell Line Genetics (Madison, WI).

## 3. Results

### 3.1. Melanoma TILs Contain More Differentiated CD8^+^ T Cells That Express High Level of PD-1 Compared with Peripheral Blood CD8^+^ T Cells

**Figure 1:**
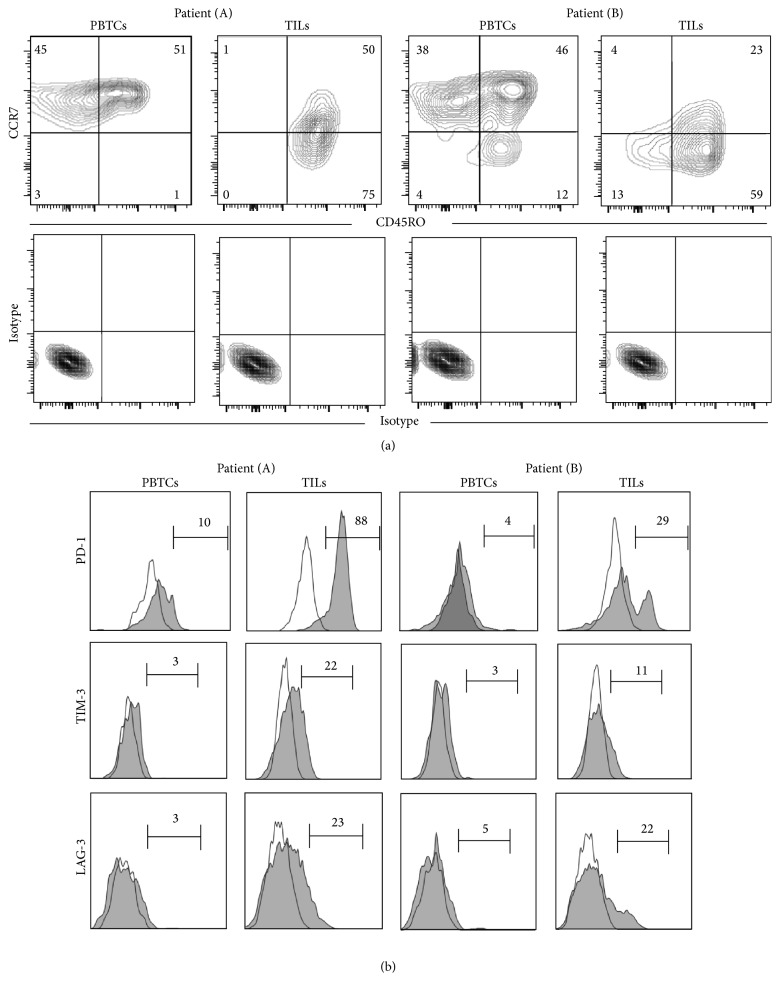
Freshly isolated CD8^+^ tumor-infiltrating lymphocytes (TILs) showed distinct phenotypical difference compared with CD8^+^ peripheral blood T cells (PBTCs). Phenotypic characterization of freshly isolated CD8^+^ T cells in TILs and PBTCs from patient (A) and patient (B). Expression of CCR7 and CD45RO (a) and PD-1, LAG-3, and TIM-3 (b) in CD8-gated live cells in TILs and PBTCs are shown. Number indicates the percentage of cells shown in each quadrant (a) or indicated gated regions (b).

Between October 2012 and March 2013, peripheral blood and tumor specimens were obtained from two adult patients who underwent resection of metastatic melanoma. We first wanted to determine the differentiation stage of T cells in the tumor and in peripheral blood which is a critical parameter influencing reprogramming potential of hematopoietic cells [25]. In both patients, assessment of T cell differentiation based on the expression of CD45RO and CCR7 showed that the majority of CD8^+^ TILs were effector memory T cells (T_EM_: CCR7^−^ CD45RO^+^) while peripheral blood T cells (PBTCs) contained proportionally more central memory T cells (T_CM_: CCR7^+^ CD45RO^+^) or stem cell memory T cells (T_SCM_: CCR7^+^ CD45RO^−^) than effector memory T cells (Figure 1(a)). This suggests freshly isolated CD8^+^ TILs from tumor would be more difficult hematopoietic cells to be reprogrammed than PBTCs based on their differentiation stage.

Recent studies have reported that CD8^+^ TILs from melanoma patients expressed high levels of inhibitory receptors such as programmed cell death protein-1 (PD-1), T cell immunoglobulin and mucin domain-3 (TIM-3), and lymphocyte activation gene-3 (LAG-3) [14–16, 28]. Of these, PD-1 expression in CD8^+^ TILs accurately identified the repertoire of clonally expanded tumor-reactive, mutation-specific lymphocytes [28]. Since our goal is to generate human iPSCs which can differentiate into patient's tumor-reactive CD8^+^ T cells for immunotherapy, we measured cell surface expression of inhibitory receptors, PD-1, TIM-3, and LAG-3. Freshly isolated CD8^+^ TILs from both patients contained higher frequencies of CD8^+^ T cells that expressed inhibitory receptors most notably PD-1 than CD8^+^ T cells in PBTCs (Figure 1(b)). Taken together, TILs from two melanoma patients in the current study contained the repertoire of clonally expanded tumor-reactive CD8^+^ T cells that were further differentiated hematopoietic cell populations than peripheral blood CD8^+^ T cells.

### 3.2. Generation of iPS Cells from Melanoma Tumor-Infiltrating Lymphocytes

Generation of iPS cells from human PBTCs was reported using Sendai virus [7, 25], lentivirus [8], or retrovirus [5, 6] that encode reprogramming factors OCT3/4, SOX2, KLF4, and c-MYC. Of these, the reprogramming efficiency was much higher with SeV system (~0.1%) compared with lentivirus or retrovirus vector (~0.001%) [6–8, 25]. Furthermore, all iPSC clones generated by using SeV system were derived from T cells while some iPSC clones generated by lentivirus or retrovirus vectors were from nonlymphoid lineages [6–8]. In addition to higher efficiency and specificity of T cell-derived iPSCs induction, SeV reprogramming system can provide integration-free iPSC clones which can avoid the possibility of insertional mutagenesis and other potential disruptions of cellular function often seen when using an integrating viral vector system for reprogramming [29]. For these reasons, we chose to use SeV system to generate iPSCs from terminally differentiated TILs.

**Figure 2:**
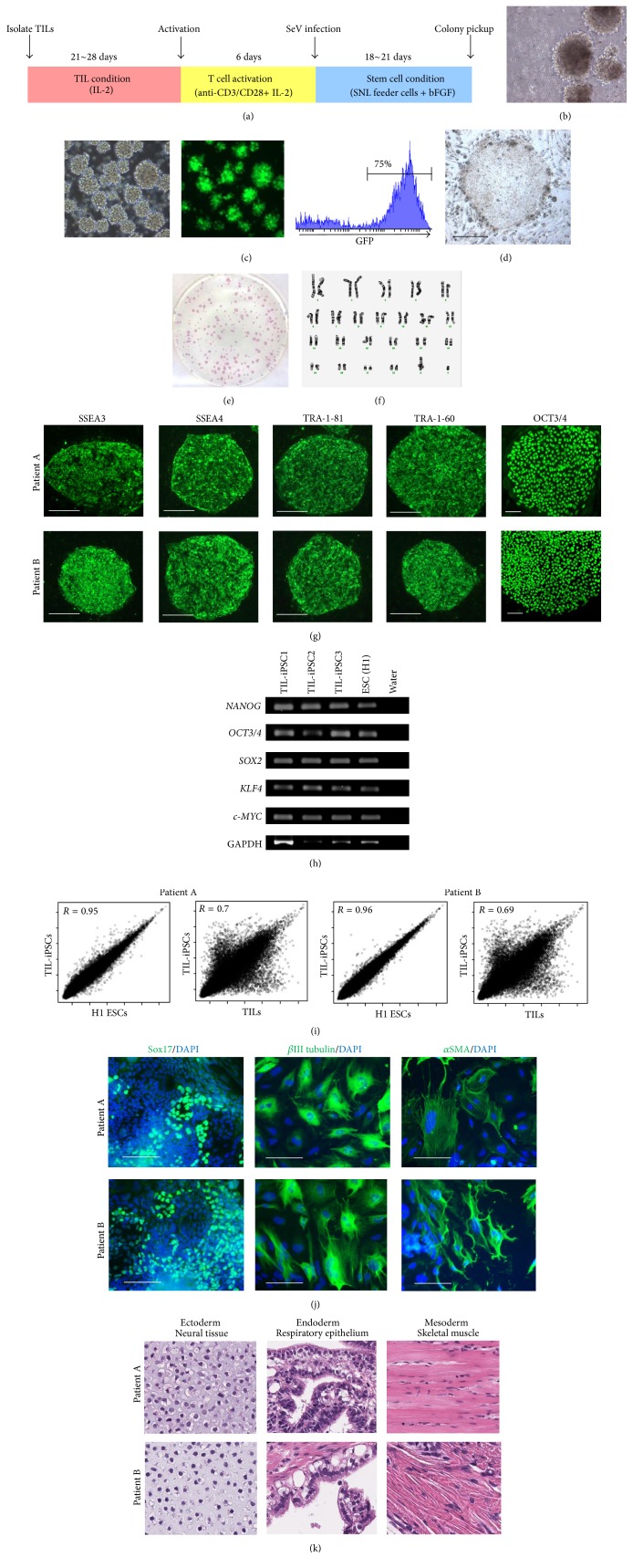
Generation of iPSCs from melanoma tumor-infiltrating lymphocytes. (a) Time schedule outlining expansion, activation, and reprogramming of TILs to generate iPSCs. (b) Morphologies of TILs when they started to expand 2-3 weeks after initiation of culture. (c) Efficient GFP introduction by Sendai virus (SeV) in TILs transfected at a MOI of 20. (d) Typical ESC-like iPSC colonies on day 21 after SeV infection. (e) Examples of 6-well plate containing SeV-reprogrammed iPSC clones stained for alkaline phosphatase (ALP), showing numerous ALP-positive colonies. (f) Cytogenetic analysis was performed on twenty G-banded metaphase cells from one of TIL-derived iPSCs (TIL-iPSCs). All twenty cells demonstrated a normal karyotype. (g) Immunofluorescence staining for pluripotency and surface markers (SSEA3, SSEA4, TRA-1-81, TRA-1-60, and OCT3/4) in iPSCs derived from melanoma TILs. Scale bars represent 100 *μ*m. (h) RT-PCR analysis for the human ES cell marker genes* NANOG*,* OCT3/4*,* SOX2*,* KLF4*, and* c-MYC* in TIL-iPSCs and ESCs (H1). (i) Scatter plots comparing the global gene expression profiles of TIL-iPSCs and ESCs and TIL-iPSCs and TILs. (j) Immunofluorescence staining for SOX17 (endodermal marker), *β*III tubulin (ectodermal marker), and *α*SMA (mesodermal marker) in TIL-iPSC-derived differentiated cell. Nuclei were counterstained with DAPI. Scale bars represent 100 *μ*m. (k) Hematoxylin and eosin-stained representative teratoma sections of TIL-iPSC clones from patients (A) and (B) (6 weeks after injection into NOD/SCID mice).

A previous report described the generation of iPSCs from PBTCs by stimulating them twice with anti-CD3 monoclonal antibody (mAb) and IL-2 5 days apart followed by infection with SeV vectors [7]. We initially used this protocol to reprogram TILs immediately after they were isolated from tumor. However, viability of the TILs was poor upon immediate stimulation with anti-CD3/CD28 mAb and IL-2. Therefore, we cultured TLs with IL-2 alone for 3-4 weeks before stimulation (Figure 2(a)). Once TILs started to expand (Figure 2(b)), they were stimulated with anti-CD3/CD28 mAb. Flow cytometric analysis of freshly isolated PBMC and TILs and TILs after 3-4 weeks of culture with IL-2 and stimulation showed the average percentage of CD8^+^ T cells was higher in freshly isolated TILs compared to PBMCs and gradually increased over the time. After first stimulation with anti-CD3/CD28 mAb, the majority of cultured cells were CD8^+^ T cells (Supplemental Figure 1). Five days after initial stimulation with anti-CD3/CD28 mAb, TILs were reactivated with anti-CD3/CD28 for 24 hr and then infected with SeV for reprogramming. First, we confirmed high transduction efficiency using SeV that encodes green fluorescent protein (SeV-GFP) (Figure 2(c)). To generate iPSCs from TILs, SeV vectors that individually encoded OCT3/4, SOX2, KLF4, and c-MYC were added to the wells at a multiplicity of infection (MOI) of 20. Twenty-four hours after gene transduction, the cells were replated onto SNL feeder cells in hESC medium containing 4 ng/mL bFGF (Figure 2(a)). Colonies that were larger and morphologically similar to hESC-like colonies were picked up within 4 weeks of infection (Figure 2(d)). Most of the colonies were positive for alkaline phosphatase (ALP) (Figure 2(e)), which is a characteristic marker of stem cells. We were able to generate ten and fifty iPSCs from patients (A) and (B), respectively. Therefore, reprogramming efficiency was 0.01% for patient (A) and 0.05% for patient (B). We found the efficiency of reprogramming PBTCs was approximately 0.1% at a MOI of 3–10 (data not shown) which is consistent with others [7, 30]. Karyotype analysis of TIL-derived iPSCs (TIL-iPSCs) showed that the cells have no chromosomal abnormalities (Figure 2(f)).

To determine whether TIL-iPSCs had the characteristics of typical hESCs, we examined stem cell marker expression. The iPSCs expressed characteristic ESC markers such as SSEA3, SSEA4, OCT3/4, TRA-1-60, and TRA-1-81 by immunofluorescence staining (Figure 2(g)). RT-PCR analysis showed that the iPSCs are positive for endogenous* NANOG*,* OCT3/4*,* SOX2*,* KLF4, *and* c-MYC* (Figure 2(h)). Global gene expression analysis confirmed that TIL-iPSCs and ESCs had similar gene expression profiles and were different from the TILs (Figure 2(i)). To further determine the pluripotency, we evaluated the differentiation potential of TIL-iPSCs by* in vitro* differentiation and teratoma formation. The TIL-iPSCs were differentiated into embryoid bodies (EBs)* in vitro*, and upregulation of marker genes for all three germ layers was detected by immunostaining (Figure 2(j)). Consistent with this* in vitro* finding, TIL-iPSCs were able to form teratomas with three germ layers such as neural tissue, respiratory epithelium, and cartilage upon subcutaneous injection into NOD/SCID mice (Figure 2(k)). Taken together, the TIL-iPSCs are pluripotent and show the molecular and morphological characteristics of human iPSCs.

### 3.3. A Wide Variety of TCR-*β* Gene Arrangement Patterns in TIL-iPSCs

**Figure 3:**
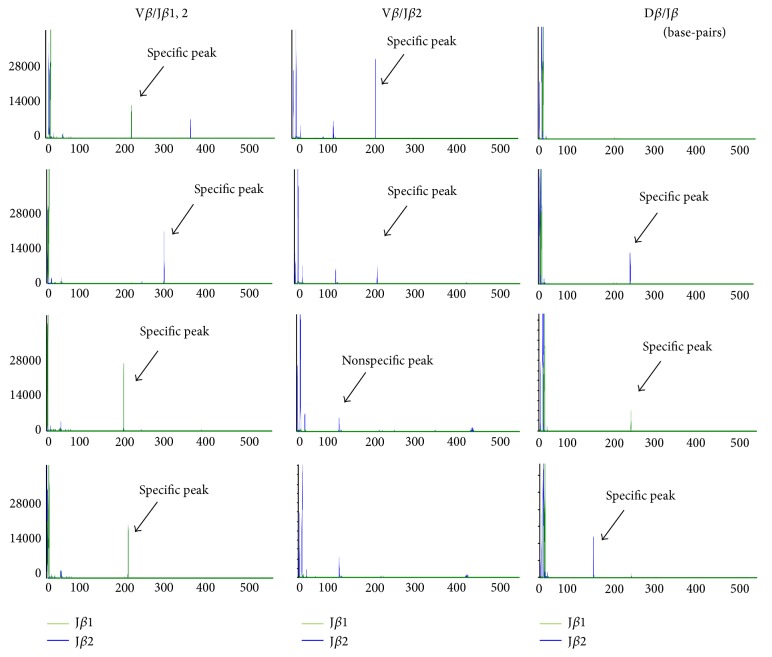
A wide variety of TCR-*β* gene arrangement patterns in TIL-iPSCs. Characterization of the TCR-*β* gene arrangement in TIL-iPSCs from patients (A) and (B) by capillary electrophoresis. The green line is derived from the band for the J*β*1 gene, and blue line is derived from the band for the J*β*2 gene.

Retained TCR rearranged gene is a hallmark of mature T cell-derived iPSCs and is carried through the differentiation to T cell lineage [5–11]. Therefore, we wanted to determine the rearrangement status of TCR-*β* genes of TIL-iPSCs to confirm that these iPSCs are derived from T cells as well as investigate TCR variability in TIL-iPSCs. We performed fragment analysis of PCR products for the genomic DNA of the TCR-*β* regions in each TIL-iPSC clone. The rearranged TCR genes were detected in all TIL-iPSCs, confirming that iPSCs were derived from T cells (Figure 3, Supplemental Figure 2). We identified 7 and 18 different TCR-*β* gene rearrangement patterns in TIL-iPSCs from patients (A) and (B), respectively.

## 4. Discussion

In the present study, we demonstrated successful reprogramming of terminally differentiated melanoma TILs that express high levels of PD-1 into human iPSCs by using SeV vectors encoding OCT3/4, SOX2, KLF4, and c-MYC. The TIL-iPSCs expressed ESC marker genes and possessed the ability to differentiate into three germ layers* in vitro* and* in vivo*. Moreover, TCR gene rearrangement analysis of TIL-iPSCs revealed that TIL-iPSCs were all derived from T cells with a wide variety of TCR rearrangement patterns, suggesting that heterogeneity of T cell clones is present in melanoma tumors.

A previous study showed that SeV reprogramming system was highly efficient and reliable in generating iPSCs not only from fibroblasts but also from peripheral blood by a single transduction with lower aneuploidy rates [30]. Our findings further extended that SeV reprogramming system was also suitable for generating iPSCs from terminally differentiated and exhausted TILs expressing high level of inhibitory receptors. Timing of stimulation with anti-CD3/CD28 mAb and infection with SeV vectors were found to be critical in reprogramming TILs. In contrast to peripheral blood T cells which can be stimulated with anti-CD3 mAb and IL-2 for reprogramming immediately after they were harvested from a donor [7], TILs needed to be cultured for 3-4 weeks with IL-2 alone before stimulation similar to the protocol used clinically for adoptive T cell therapy [26, 31].

Our study demonstrated that melanoma TILs from two patients expressed high level of PD-1, which is consistent with reports from others [14–16]. A recent study from Gros et al. further demonstrated expression of PD-1 on CD8^+^ melanoma TILs identified the repertoire of clonally expanded tumor-reactive, mutation-specific lymphocytes and these populations played a critical role in tumor regression after adoptive cell therapy [28]. Our data of reprogramming TILs expressing high level of PD-1 indicates successful generation of iPSCs from patient-specific tumor-reactive CD8^+^ T cells. However, this needs to be further confirmed by evaluating the antitumor reactivity of TIL-iPSC-derived T cells against the patient's tumor cells* in vitro* and/or* in vivo* using a xenograft model [9, 10, 13].

We found TILs displayed more differentiated phenotype and lower reprogramming efficiency compared to peripheral blood T cells, which is in line with prior observations that differentiation stage has a strong impact on the efficiency of reprogramming hematopoietic cells into iPSCs [25]. Another possible explanation is that cells being in an exhausted state have lower reprogramming efficiency. The difference of PD-1 expression and reprogramming efficiency of TILs between patients (A) and (B) might suggest cells being in an exhausted state may have a major influence on the efficiency of reprogramming into iPSCs. However, this possibility needs to be further evaluated with more cases.

Recent significant progress made in reprogramming technology might improve reprogramming efficiency for generation of TIL-iPSCs. A new SeV vector, TS12KOS, was found to have improved efficiency of iPSC generation compared with the conventional SeV vectors used in the current study [32, 33]. Another promising avenue for future investigation is to combine gene transduction with signal inhibition or chemical compounds which was found to enhance the efficiency of iPSC generation [34–39]. Furthermore, Rais et al. recently reported that depletion of Mbd3 (methyl-CpG binding domain protein 3) combined with induction of the Yamanaka factors (OCT3/4, SOX2, KLF4, and c-MYC) significantly enhanced reprogramming efficiency and achieved nearly 100% complete conversion of mouse embryonic fibroblasts to pluripotency within a week [40].

In line with previous observations in PBTC-derived iPSCs [7], our data demonstrated that all iPSCs generated by SeV reprogramming system were derived from T cells. While human iPSCs derived from fibroblast are capable of producing T lymphocyte populations with a broad TCR repertoire [41], T cell-derived iPSCs bearing rearranged TCR genes differentiate into T lineage expressing the same TCR from the original T cells [9–11]. This will allow rejuvenated TILs to retain the antigen specificity after reprogramming and redifferentiation. A wide variety of TCR rearrangement patterns in TIL-iPSC clones suggests heterogeneity of T cell clones in melanoma tumors. This result is in support of the study from Yazdi et al., who used laser-capture microdissection to isolate different TIL clusters from melanoma and demonstrated that many different T cell clones with different rearrangements were detected within the same individual [42].

Although the antitumor efficacy of TIL-iPSCs or TIL-iPSC-derived T cells remains to be elucidated, recent findings from several groups suggest therapeutic potential of iPSC-derived T cells. Vizcardo et al. reported successful reprogramming of a T cell clone recognizing the MART-1 antigen expressed on melanoma tumors and* in vitro* antitumor immunity of regenerated MART-1 iPSC-derived T cells [10]. Nishimura et al. showed generation of iPSCs from HIV-1-epitope specific clone of CD8^+^ T cells and regeneration of functional antigen-specific T cells with capacity of producing cytokine and cytolytic activity [9]. Themeli et al. provided another insight into whether this approach can be used in combination with chimeric antigen receptor (CAR) technology by showing that genetically engineered T cell-derived iPSCs to express a CAR specific to the CD19 antigen can differentiate into CAR-expressing T cells which have antitumor reactivity against CD19^+^ lymphoma* in vitro* and* in vivo* [13].

Potential advantage of reprogramming and regeneration of TILs would be to obtain polyclonal antitumor T cells targeting heterogeneous populations of solid tumors for cancer immunotherapy. Recent advances in sequencing analyses of solid malignancies have identified significant heterogeneity between tumors as well as within an individual tumor, which can contribute to treatment failure and drug resistance [43–46]. Our ultimate goal is to differentiate TIL-iPSCs with a wide variety of TCR gene rearrangement sequences into T cells and determine their antitumor reactivity against heterogeneous tumor cell populations. Of note, this strategy can also be used not only for the known tumor antigens such as CD19 or MART-1 but also for unknown tumor antigens once antitumor reactivity of each TIL-iPSC-derived T cell is identified.

This strategy has some limitations in addition to the costs and labor intensity of generating TIL-iPSCs from each patient. It takes significant amount of time to expand TILs, generate iPSCs, differentiate iPSCs to T cells, and identify their tumor specificity. One possible way to utilize this approach would be to identify the TIL-iPSCs that differentiate into tumor-specific T cells and store the TIL-iPSCs for other patients with cancers expressing the appropriate tumor antigen(s) and HLA haplotypes, which has been described for virus-specific T cells [47, 48] or pluripotent stem cells [49–52]. These iPSCs bearing rearranged TCR of known antigen specificity can differentiate into tumor-specific T cells* in vivo* to promote cancer immunosurveillance or* in vitro* to generate an infinite number of phenotypically defined, functional, and expandable polyclonal T cells for adoptive T cell therapy [12, 13].

## Supplementary Material

Flow cytometry analysis of freshly isolated PBMCs and TILs, and TILs after 3-4 weeks of culture with IL-2 and stimulation are presented in Supplemental figure 1. TILs were cultured with IL-2 only for 3-4 weeks, then stimulated twice with anti-CD3/CD28 and IL-2 for reprogramming. Data are represented as the percentage of NK1.1^+^ cells, CD4^+^ T cells, CD8^+^ T cells and CD19^+^ B cells. Data are representative of two independently performed experiments. Characterization of the TCR-β gene arrangement in TIL-iPSC clones by capillary electrophoresis associated with figure 3 are presented in Supplemental figure2. The green line is derived from the band for the Jβ1 gene, and blue line is derived from the band for the Jβ2 gene. In the left, middle and right panel, rearrangement of Vβ/Jβ1, 2 region, Vβ/Jβ2 region and Dβ/ Jβ is shown, respectively. TIL-iPSC clone A1-A7 were derived from patient (A) and TIL-iPSC clone B1-18 were from patient (B). All TIL-iPSC clones showed different TCR-β rearrangement pattern. Primers used in gene expression analysis by Reverse Transcription Polymerase Chain Reaction (RT-PCR) are shown in Table S1.

## References

[1] Takahashi K., Yamanaka S. (2006). Induction of pluripotent stem cells from mouse embryonic and adult fibroblast cultures by defined factors. *Cell*.

[2] Takahashi K., Tanabe K., Ohnuki M. (2007). Induction of pluripotent stem cells from adult human fibroblasts by defined factors. *Cell*.

[3] Yu J., Vodyanik M. A., Smuga-Otto K. (2007). Induced pluripotent stem cell lines derived from human somatic cells. *Science*.

[4] Park I.-H., Zhao R., West J. A. (2008). Reprogramming of human somatic cells to pluripotency with defined factors. *Nature*.

[5] Brown M. E., Rondon E., Rajesh D. (2010). Derivation of induced pluripotent stem cells from human peripheral blood T lymphocytes. *PLoS ONE*.

[6] Loh Y.-H., Hartung O., Li H. (2010). Reprogramming of T cells from human peripheral blood. *Cell Stem Cell*.

[7] Seki T., Yuasa S., Oda M. (2010). Generation of induced pluripotent stem cells from human terminally differentiated circulating T cells. *Cell Stem Cell*.

[8] Staerk J., Dawlaty M. M., Gao Q. (2010). Reprogramming of human peripheral blood cells to induced pluripotent stem cells. *Cell Stem Cell*.

[9] Nishimura T., Kaneko S., Kawana-Tachikawa A. (2013). Generation of rejuvenated antigen-specific T cells by reprogramming to pluripotency and redifferentiation. *Cell Stem Cell*.

[10] Vizcardo R., Masuda K., Yamada D. (2013). Regeneration of human tumor antigen-specific T cells from iPSCs derived from mature CD8^+^ T cells. *Cell Stem Cell*.

[11] Wakao H., Yoshikiyo K., Koshimizu U. (2013). Expansion of functional human mucosal-associated invariant T cells via reprogramming to pluripotency and redifferentiation. *Cell Stem Cell*.

[12] Lei F., Zhao B., Haque R. (2011). In vivo programming of tumor antigen-specific T lymphocytes from pluripotent stem cells to promote cancer immunosurveillance. *Cancer Research*.

[13] Themeli M., Kloss C. C., Ciriello G. (2013). Generation of tumor-targeted human T lymphocytes from induced pluripotent stem cells for cancer therapy. *Nature Biotechnology*.

[14] Inozume T., Hanada K.-I., Wang Q. J. (2010). Selection of CD8+PD-1+ lymphocytes in fresh human melanomas enriches for tumor-reactive T cells. *Journal of Immunotherapy*.

[15] Ahmadzadeh M., Johnson L. A., Heemskerk B. (2009). Tumor antigen-specific CD8 T cells infiltrating the tumor express high levels of PD-1 and are functionally impaired. *Blood*.

[16] Baitsch L., Baumgaertner P., Devêvre E. (2011). Exhaustion of tumor-specific CD8^+^ T cells in metastases from melanoma patients. *Journal of Clinical Investigation*.

[17] Gattinoni L., Klebanoff C. A., Palmer D. C. (2005). Acquisition of full effector function in vitro paradoxically impairs the in vivo antitumor efficacy of adoptively transferred CD8^+^ T cells. *The Journal of Clinical Investigation*.

[18] Yee C., Thompson J. A., Byrd D. (2002). Adoptive T cell therapy using antigen-specific CD8^+^ T cell clones for the treatment of patients with metastatic melanoma: In vivo persistence, migration, and antitumor effect of transferred T cells. *Proceedings of the National Academy of Sciences of the United States of America*.

[19] Zhou J., Shen X., Huang J., Hodes R. J., Rosenberg S. A., Robbins P. F. (2005). Telomere length of transferred lymphocytes correlates with in vivo persistence and tumor regression in melanoma patients receiving cell transfer therapy. *Journal of Immunology*.

[20] Dudley M. E., Gross C. A., Langhan M. M. (2010). CD8+ enriched ‘Young’ tumor infiltrating lymphocytes can mediate regression of metastatic melanoma. *Clinical Cancer Research*.

[21] Berger C., Jensen M. C., Lansdorp P. M., Gough M., Elliott C., Riddell S. R. (2008). Adoptive transfer of effector CD8^+^ T cells derived from central memory cells establishes persistent T cell memory in primates. *Journal of Clinical Investigation*.

[22] Klebanoff C. A., Gattinoni L., Torabi-Parizi P. (2005). Central memory self/tumor-reactive CD8^+^ T cells confer superior antitumor immunity compared with effector memory T cells. *Proceedings of the National Academy of Sciences of the United States of America*.

[23] Gattinoni L., Lugli E., Ji Y. (2011). A human memory T cell subset with stem cell-like properties. *Nature Medicine*.

[24] Blelloch R., Wang Z., Meissner A., Pollard S., Smith A., Jaenisch R. (2006). Reprogramming efficiency following somatic cell nuclear transfer is influenced by the differentiation and methylation state of the donor nucleus. *Stem Cells*.

[25] Eminli S., Foudi A., Stadtfeld M. (2009). Differentiation stage determines potential of hematopoietic cells for reprogramming into induced pluripotent stem cells. *Nature Genetics*.

[26] Dudley M. E., Wunderlich J. R., Shelton T. E., Even J., Rosenberg S. A. (2003). Generation of tumor-infiltrating lymphocyte cultures for use in adoptive transfer therapy for melanoma patients. *Journal of Immunotherapy*.

[27] van Dongen J. J. M., Langerak A. W., Brüggemann M. (2003). Design and standardization of PCR primers and protocols for detection of clonal immunoglobulin and T-cell receptor gene recombinations in suspect lymphoproliferations: report of the BIOMED-2 concerted action BMH4-CT98-3936. *Leukemia*.

[28] Gros A., Robbins P. F., Yao X. (2014). PD-1 identifies the patient-specific CD8^+^ tumor-reactive repertoire infiltrating human tumors. *The Journal of Clinical Investigation*.

[29] Genovese P., Schiroli G., Escobar G. (2014). Targeted genome editing in human repopulating haematopoietic stem cells. *Nature*.

[30] Schlaeger T. M., Daheron L., Brickler T. R. (2015). A comparison of non-integrating reprogramming methods. *Nature Biotechnology*.

[31] Rosenberg S. A., Yang J. C., Sherry R. M. (2011). Durable complete responses in heavily pretreated patients with metastatic melanoma using T-cell transfer immunotherapy. *Clinical Cancer Research*.

[32] Ban H., Nishishita N., Fusaki N. (2011). Efficient generation of transgene-free human induced pluripotent stem cells (iPSCs) by temperature-sensitive Sendai virus vectors. *Proceedings of the National Academy of Sciences of the United States of America*.

[33] Fujie Y., Fusaki N., Katayama T. (2014). New type of sendai virus vector provides transgene-free iPS cells derived from chimpanzee blood. *PLoS ONE*.

[34] Liang G., Taranova O., Xia K., Zhang Y. (2010). Butyrate promotes induced pluripotent stem cell generation. *The Journal of Biological Chemistry*.

[35] Zhu S., Li W., Zhou H. (2010). Reprogramming of human primary somatic cells by OCT4 and chemical compounds. *Cell Stem Cell*.

[36] Huangfu D., Maehr R., Guo W. (2008). Induction of pluripotent stem cells by defined factors is greatly improved by small-molecule compounds. *Nature Biotechnology*.

[37] Li W., Wei W., Zhu S. (2009). Generation of rat and human induced pluripotent stem cells by combining genetic reprogramming and chemical inhibitors. *Cell Stem Cell*.

[38] Silva J., Barrandon O., Nichols J., Kawaguchi J., Theunissen T. W., Smith A. (2008). Promotion of reprogramming to ground state pluripotency by signal inhibition. *PLoS Biology*.

[39] Maherali N., Hochedlinger K. (2009). Tgf*β* signal inhibition cooperates in the induction of iPSCs and replaces Sox2 and cMyc. *Current Biology*.

[40] Rais Y., Zviran A., Geula S. (2013). Deterministic direct reprogramming of somatic cells to pluripotency. *Nature*.

[41] Chang C.-W., Lai Y.-S., Lamb L. S., Townes T. M. (2014). Broad T-cell receptor repertoire in T-lymphocytes derived from human induced pluripotent stem cells. *PLoS ONE*.

[42] Yazdi A. S., Morstedt K., Puchta U. (2006). Heterogeneity of T-cell clones infiltrating primary malignant melanomas. *Journal of Investigative Dermatology*.

[43] Menzies A. M., Haydu L. E., Carlino M. S. (2014). Inter- and intra-patient heterogeneity of response and progression to targeted therapy in metastatic melanoma. *PLoS ONE*.

[44] Sjöblom T., Jones S., Wood L. D. (2006). The consensus coding sequences of human breast and colorectal cancers. *Science*.

[45] Gerlinger M., Rowan A. J., Horswell S. (2012). Intratumor heterogeneity and branched evolution revealed by multiregion sequencing. *The New England Journal of Medicine*.

[46] Mullighan C. G., Phillips L. A., Su X. (2008). Genomic analysis of the clonal origins of relapsed acute lymphoblastic leukemia. *Science*.

[47] Gallot G., Vollant S., Saïagh S. (2014). T-cell therapy using a bank of EBV-specific cytotoxic T cells: lessons from a phase I/II feasibility and safety study. *Journal of Immunotherapy*.

[48] Gourraud P.-A., Gilson L., Girard M., Peschanski M. (2012). The role of human leukocyte antigen matching in the development of multiethnic ‘haplobank’ of induced pluripotent stem cell lines. *Stem Cells*.

[49] Leen A. M., Bollard C. M., Mendizabal A. M. (2013). Multicenter study of banked third-party virus-specific T cells to treat severe viral infections after hematopoietic stem cell transplantation. *Blood*.

[50] Nakatsuji N., Nakajima F., Tokunaga K. (2008). HLA-haplotype banking and iPS cells. *Nature Biotechnology*.

[51] Stacey G. N., Crook J. M., Hei D., Ludwig T. (2013). Banking human induced pluripotent stem cells: lessons learned from embryonic stem cells?. *Cell Stem Cell*.

[52] Turner M., Leslie S., Martin N. G. (2013). Toward the development of a global induced pluripotent stem cell library. *Cell Stem Cell*.

