# Supplementary material for: Reprogramming of Melanoma Tumor-Infiltrating Lymphocytes to Induced Pluripotent Stem Cells
Source: Stem Cells Int. 2015 Dec 28;2016:8394960. doi: 10.1155/2016/8394960 (PMC4707343; doi:10.1155/2016/8394960)
Supplement: Supplementary file 1 — Flow cytometry analysis of freshly isolated PBMCs and TILs, and TILs after 3-4 weeks of culture with IL-2 and stimulation are presented in Supplemental figure 1. TILs were cultured with IL-2 only for 3-4 weeks, then stimulated twice with anti-CD3/CD28 and IL-2 for reprogramming. Data are represented as the percentage of NK1.1+ cells, CD4+ T cells, CD8+ T cells and CD19+ B cells. Data are representative of two independently performed experiments. Characterization of the TCR-β gene arrangement in TIL-iPSC clones by capillary electrophoresis associated with figure 3 are presented in Supplemental figure2. The green line is derived from the band for the Jβ1 gene, and blue line is derived from the band for the Jβ2 gene. In the left, middle and right panel, rearrangement of Vβ/Jβ1, 2 region, Vβ/Jβ2 region and Dβ/ Jβ is shown, respectively. TIL-iPSC clone A1-A7 were derived from patient (A) and TIL-iPSC clone B1-18 were from patient (B). All TIL-iPSC clones showed different TCR-β rearrangement pattern. Primers used in gene expression analysis by Reverse Transcription Polymerase Chain Reaction (RT-PCR) are shown in Table S1. [file 8394960.f1.pdf]

**Supplemental Figure 1**

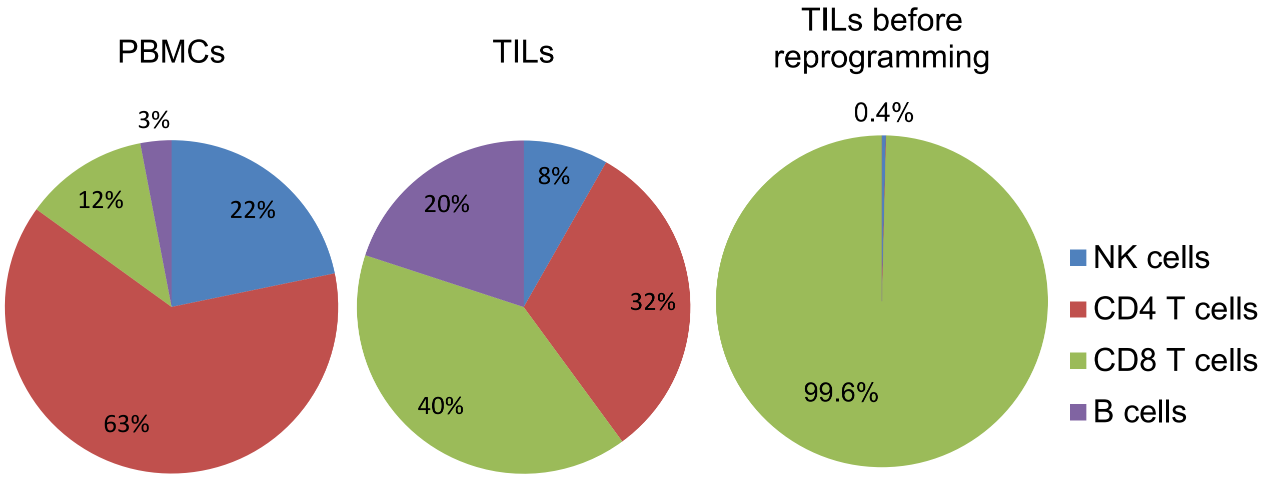

## Supplemental Figure 2

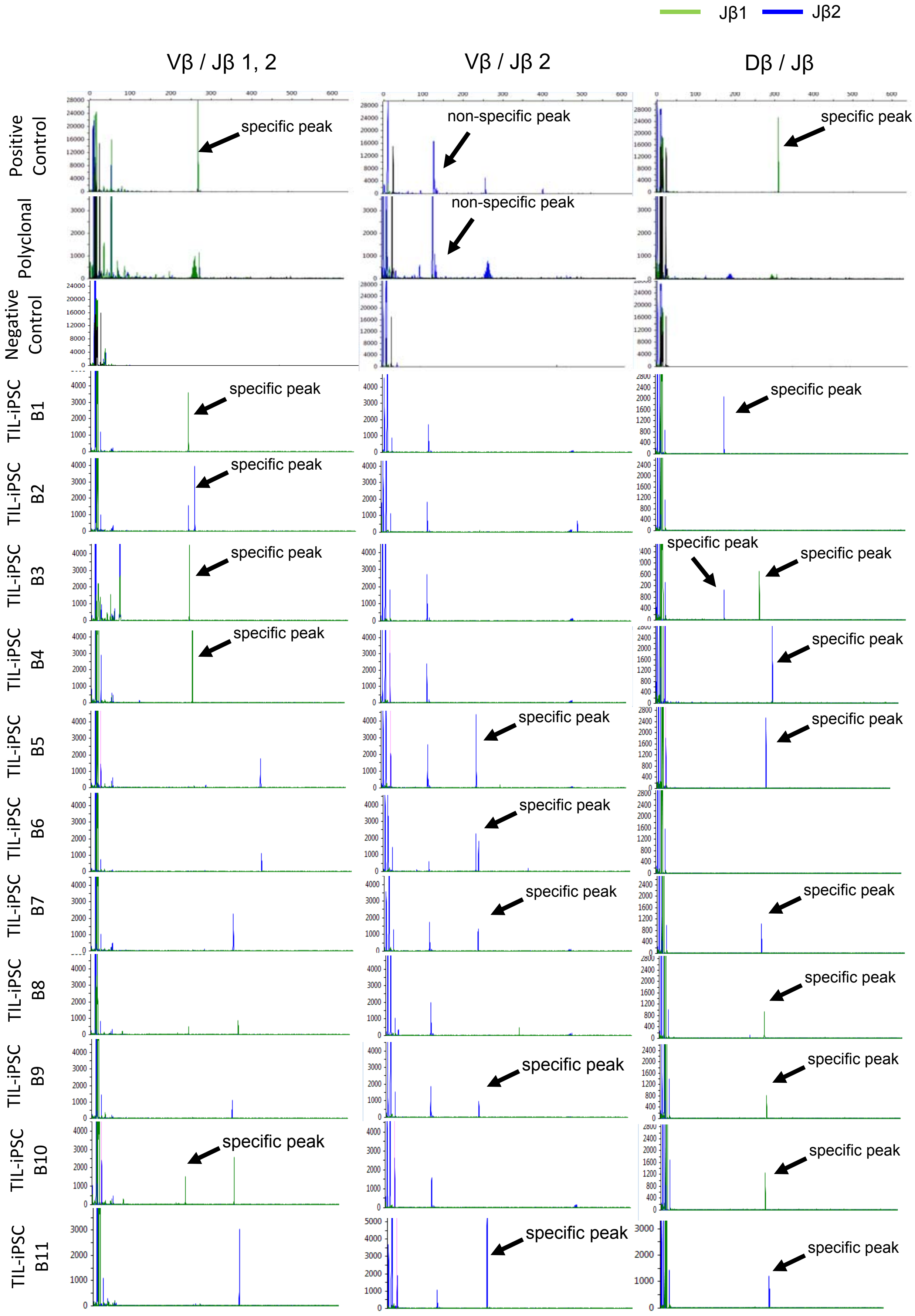

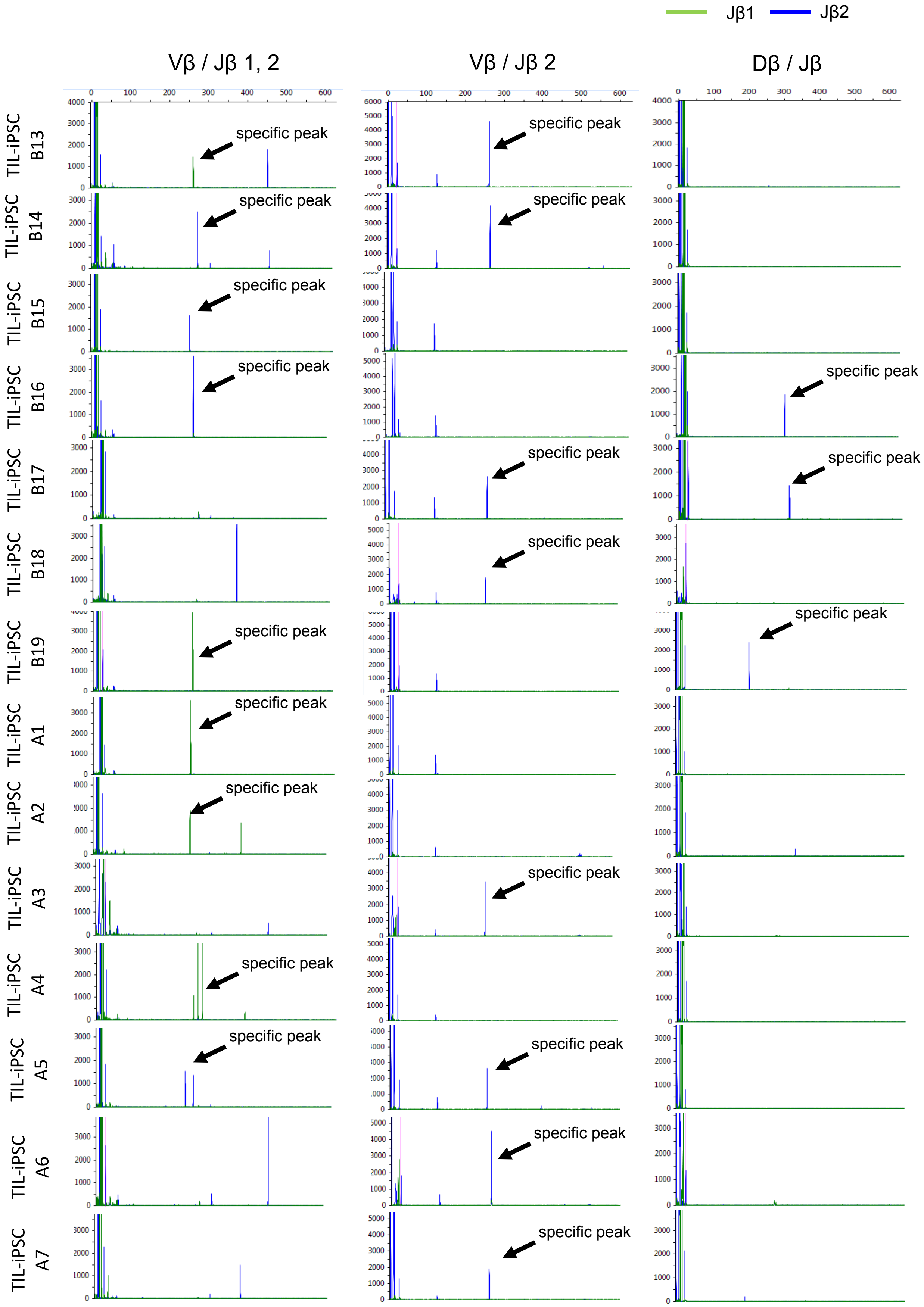
Jβ1
Jβ2
Vβ / Jβ 1, 2
Vβ / Jβ 2
Dβ / Jβ
TIL-iPSC B13
TIL-iPSC B14
TIL-iPSC B15
TIL-iPSC B16
TIL-iPSC B17
TIL-iPSC B18
TIL-iPSC B19
TIL-iPSC A1
TIL-iPSC A2
TIL-iPSC A3
TIL-iPSC A4
TIL-iPSC A5
TIL-iPSC A6
TIL-iPSC A7
specific peak

**Table S1**

The sequences of primer sets

| Genes | Sequences (Forward: F, Reverse: R) |
|---|---|
| OCT3/4 | F: AGTCAGTGAACAGGGAATGG |
| | R: TCGGGATTCAAGAACCTACG |
| SOX2 | F: GAGAGAAAGAAAGGGAGAGAAG |
| | R: GAGAGAGGCAAACTGGAATC |
| KLF4 | F: GATGAACTGACCAGGCACTA |
| | R: GTGGGTCATATCCACTGTCT |
| cMYC | F: TGCCTCAAATTGGACTTTGG |
| | R: GATTGAAATTCTGTGTAACTGC |
| NANOG | F: TCCTCCTCTTCCTCTATACTAAC |
| | R: CCCACAAATCACAGGCATAG |
| GAPDH | F: TGAAGGTCGGAGTCAACGGATTTGGT |
| | R: CATGTGGGCCATGAGGTCCACCAC |
